# Strain Transfer Mechanism in Surface-Bonded Distributed Fiber Optic Sensors under Different Strain Fields

**DOI:** 10.3390/s23156863

**Published:** 2023-08-02

**Authors:** Wenbo Du, Xing Zheng, Bin Shi, Mengya Sun, Hao Wu, Weida Ni, Zhenming Zheng, Meifeng Niu

**Affiliations:** 1 POWERCHINA Huadong Engineering Corporation Limited, Hangzhou 311122, China; du_wb@hdec.com (W.D.); ni_wd@hdec.com (W.N.); 2 College of Engineering, Ocean University of China, Qingdao 266100, China; 3 Zhejiang Huadong Geotechnical Investigation & Design Institute Corporation Limited, Hangzhou 310030, China; wu_h5@hdec.com (H.W.); zheng_zm5@hdec.com (Z.Z.); niu_mf@hdec.com (M.N.); 4 School of Earth Sciences and Engineering, Nanjing University, Nanjing 210046, China; shibin@nju.edu.cn; 5 School of Earth Sciences and Engineering, Hohai University, Nanjing 211000, China

**Keywords:** strain transfer, distributed fiber optic sensor, shear lag, strain-sensing cable, optical frequency-domain reflectometer, strain field, monitored host

## Abstract

Mastering the strain transfer mechanism in distributed fiber optic (DFO) sensors holds the key to analyzing strain measurement errors from DFO sensing systems. However, the impact of the monitored structure’s strain distribution on the strain transfer mechanism in DFO sensors has often been overlooked in the existing research. To address this issue, a strain transfer model of surface-bonded DFO sensors with multilayered structures was established based on the shear lag theory. The closed-form solutions of the strain transfer coefficient of DFO sensors subjected to uniform, parabolic, single-linear gradient, and bilinear gradient strains were obtained. With a high-accuracy optical frequency-domain reflectometer (OFDR), the theoretical model was validated by laboratory tests. Upon parametric analysis, suggestions were further offered about designing and installing DFO sensors.

## 1. Introduction

Distributed fiber optic (DFO) sensing, characterized by a long sensing distance, easy implantation, long-term stability, and high accuracy [1], is widely used in the field monitoring of engineering structures [2,3,4], geotechnical engineering [5,6,7,8], and the geo-environment [9,10,11,12,13,14,15]. DFO sensors act as sensing nerves in DFO sensing systems [16,17,18], acquiring real-time, multi-physical field information such as the temperature and strain of the monitored object [19,20,21]. Notably, the conventional sensing optical fiber, as delicate as a hair strand, has difficulty surviving the harsh environment of practical applications. Thus, DFO sensing fibers necessitate multilayered packaging and are made into sensing cables to increase their viability [22]. However, strain loss occurs when the strain is transferred from the monitored host toward the fiber core, resulting in strain measurement errors. As such, it is important to master the strain transfer mechanism of DFO sensor for error analysis and correction of monitoring results [23].

In the early 1990s, the strain transfer between fiber optic (FO) sensors and the monitored host drew the attention of researchers. In the monitoring of concrete structures, Nanni et al. [24] determined the strain transfer coefficient between the structure and FO sensor, which was found to increase as the Young’s modulus of the cable protective layer came closer to that of the fiber core. Before long, Ansari and Yuan [25] developed a strain transfer model for embedded FO sensors in a uniform strain field based on shear lag theory proposed by Cox [26]. They also observed the strain transfer in a dual-layered fiber Bragg grating (FBG), a move that underpins subsequent engineering practices and theoretical analyses. As a way to modify the model introduced by Ansari and Yuan [25], Li [27] raised a hypothesis that the strain gradient of midpoints in each layer of the FO sensor is approximately equal, suggesting a closer alignment between the results of strain transfer analysis and experiments. On this basis, Li [28] analyzed the strain transfer of a FBG under non-axial uniform strain. Since then, the research into strain transfer theory has gradually developed from a FBG to a sensing cable with multilayered structures. Feng et al. [29] deduced the strain transfer model using a polar coordinate system and analyzed the strain transfer phenomenon of the FO cable used in crack detection. By introducing the Goodman hypothesis and considering the properties of matrix materials around the optical fiber, Wang et al. [30] analyzed the effects of the viscoelasticity of matrix materials and ambient temperature on the strain transfer coefficient. Thanks to the development of the high-precision optical frequency-domain reflectometer (OFDR), Zhang et al. [31] systematically studied the effects of the mechanical parameters and bonding methods of an optical cable on optical cable strain transfer through a series of experiments. Falcetelli et al. [32] established a strain transfer model of a surface-bonded FO cable with multilayered structures, deduced the strain transfer coefficient of a surface-adhesive optical fiber sensor when the boundary condition was not 0, and compared the experimental results with the model in reference [27], which proved the effectiveness of the model and enriched the theoretical system of strain transfer.

However, the previous studies invariably considered the strain of the monitored host as uniform, disregarding the strain field’s impact on the strain transfer performance of FO sensing cables. Such a hypothesis also fails to take into account the complex stress characteristics of engineering structures. To bridge this gap, Zhang et al. [33] delved into the peculiarities of parabolic strain distribution within the monitored host and the slippage at the host–cable sheath interface and deduced analytical solutions for the strain transfer coefficients of the cable. Their calculations, however, remain to be validated. Following that, Zhang [34] derived the strain transfer coefficient of the cable under the scenarios in which the monitored host was subjected to a uniform force or concentrated force, before verifying the theoretical model using the finite element method and model-based testing. However, given the constrained spatial resolution of the Brillouin optical time-domain analysis (BOTDA) technique, the experimental results fell short of expectations. Centering on the concentrated force scenario, Zheng et al. [35] worked out the strain transfer coefficients of the FO cable featuring single-linear and bilinear gradient strain distributions within the host. Additionally, they tested and validated their proposed model using OFDR, but neglected the strain loss at the inflection point of the bilinear gradient strain field by regarding the point’s strain transfer coefficient as 1. Alternatively, Tan et al. [36], assuming continuity between the strain and shear stress functions at the inflection point, demonstrated the existence of strain loss at the said inflection point, thus providing novel insights into the analysis of strain transfer mechanisms for sensing cables within continuous, non-uniform strain fields.

Overall, despite the great scholarly attention given to the strain transfer mechanism in FO sensors, how DFO sensors are influenced by the strain distribution of the monitored host awaits systematic research. Expanding on the prior research, a theoretical strain transfer model for surface-bonded DFO sensors with multilayered structures was established in this paper. The closed-form analytical solutions for the strain transfer coefficients of a cable with strain distributed in uniform, parabolic, single-linear gradient, and bilinear gradient manners were derived. Subsequently, the proposed model and solutions were validated by a laboratory test. As a theoretical foundation for strain transfer analysis in FO sensors within complex strain fields, this study also serves as a guide to the design and installation of FO sensors.

## 2. Strain Transfer Model

**Figure 1:**
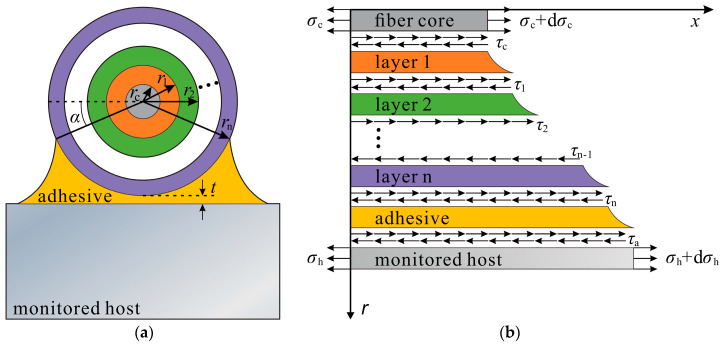
Strain transfer mechanism for the surface-bonded FO sensing cable with multilayered structures: (**a**) Cross-section; (**b**) Stress state of cable elements.

Taking the surface-bonded DFO sensor as an example and based on geometric modeling as provided by Falcetelli et al. [32], a strain transfer model of surface-bonded DFO sensing cables with multilayered structures was established. The model, as shown in Figure 1, follows three hypotheses:

(1) The core, protective layers (jacket), and the adhesive layer of the FO cable are linear elastic materials, with each layer well-bonded and featuring no relative slippage.
(2) Made from silicon dioxide, the fiber core and cladding are collectively known as the fiber core layer.
(3) Only the shear stress transfer between each protective layer and adhesive within the bonded area is factored in.

The model was established in the polar coordinate system, where *x* is the axial position of the optical fiber; *r* represents the fiber’s radial position; *θ* indicates an angle. By reference to Figure 1b, the equation of mechanical equilibrium for elements in the fiber core layer can be expressed as:

(1) $$(\sigma_{c}+d\sigma_{c})\pi r_{c}{}^{2}-\sigma_{c}\pi r_{c}{}^{2}+\int_{0}^{2\pi }\tau (x,r_{c})r_{c}d\theta \cdot dx=0$$

where *r*_c_ is the fiber core layer’s radius; $\sigma_{c}$ represents the normal stress across the fiber core’s cross-section; $\tau (x,r_{c})$ indicates the shear stress at the interface between the fiber core and the first jacket.

Reducing Equation (1) gives:

(2) $$\tau (x,r_{c})=-\frac{r_{c}}{2}\frac{d\sigma_{c}}{dx}$$

In light of Hypothesis (3), the mechanical equilibrium equation for the first jacket can be expressed as:

(3) $$\int_{\alpha }^{\pi -\alpha }\tau (x,r)rd\theta \cdot dx-\int_{0}^{2\pi }\tau (x,r_{c})rd\theta \cdot dx=0$$

where α is the angle between the horizontal line and the boundary between the adhesive and the bonded area. The equation for the shear stress at the interface between the first and second jackets is obtained by writing out the set of Equations (2) and (3):

(4) $$\tau (x,r)=-\frac{\pi }{\pi -2\alpha }\frac{r_{c}{}^{2}}{r}\frac{d\sigma_{c}}{dx}$$

According to Hypothesis (1) that the fiber core and each protective layer are linear elastic materials, and to Hooke’s law, the shear strain at the interface between the first and second jackets can be expressed as:

(5) $$\gamma (x,r)=-\frac{1}{G_{1}}\frac{\pi }{\pi -2\alpha }\frac{r_{c}{}^{2}}{r}E_{c}\frac{d\varepsilon_{c}}{dx}$$

where γ(x,r) represents the shear strain at the interface between the first and second jackets; $G_{1}$ is the shear modulus of the first jacket; *E*_c_ denotes the elastic modulus of the fiber core layer; $\varepsilon_{c}$ indicates the strain of the fiber core layer.

Given that radial displacement is far smaller than axial displacement, we obtained:

(6) $$\gamma (x,r)≅\frac{\partial u}{\partial r}=-\frac{1}{G_{1}}\frac{\pi }{\pi -2\alpha }\frac{r_{c}{}^{2}}{r}E_{c}\frac{d\varepsilon_{c}}{dx}$$

where *u* is axial displacement. An integration of Equation (6) yields the axial displacement at the boundary of the first jacket:

(7) $$\int_{r_{c}}^{r_{1}}\frac{\partial u}{\partial r}=\int_{r_{c}}^{r_{1}}-\frac{1}{G_{1}}\frac{\pi }{\pi -2\alpha }\frac{r_{c}{}^{2}}{r}E_{c}\frac{d\varepsilon_{c}}{dx}dr$$

(8) $$u_{1}-u_{c}=-\frac{1}{G_{1}}\frac{\pi }{\pi -2\alpha }r_{c}{}^{2}E_{c}\frac{d\varepsilon_{c}}{dx}\ln\frac{r_{1}}{r_{c}}$$

where $u_{1}$ and $u_{c}$ represent the axial displacement at the boundary of the first cable jacket and of the fiber core layer, respectively.

The axial displacement at the boundary of the remaining protective layers and of the adhesive layer can be inferred in this manner, thus giving:

(9) $$u_{h}-u_{c}=-\frac{\pi }{\pi -2\alpha }r_{c}{}^{2}E_{c}\frac{d\varepsilon_{c}}{dx}\left[\frac{1}{G_{a}}\ln\frac{r_{a}}{r_{n}}+\frac{1}{G_{n}}\ln\frac{r_{n}}{r_{n-1}}+\ldots +\frac{1}{G_{2}}\ln\frac{r_{2}}{r_{1}}+\frac{1}{G_{1}}\ln\frac{r_{1}}{r_{c}}\right]$$

where $u_{h}$ is the axial displacement at the adhesive–host interface; $r_{1}∼r_{n}$ and $G_{1}∼G_{n}$ are the radius and shear modulus of Jacket 1 to Jacket n, respectively; $G_{a}$ is the adhesive layer’s shear modulus; $r_{a}$ indicates the adhesive layer’s equivalent radius, which, based on the model’s geometrical characteristics, can be calculated with Equation (10):

(10) $$r_{a}=\frac{1}{\pi -2\alpha }\int_{\alpha }^{\pi -\alpha }\left[r_{n}(1-\sin\alpha )+t\right]d\theta =r_{n}+t-\frac{2r_{c}\cos\alpha }{\pi -2\alpha }$$

where *t* denotes the thickness of the adhesive layer from the bottom of the cable to the monitored host’s surface, as shown in Figure 1a. As the shear lag coefficient *k* is introduced, Equation (9) can be reduced to:

(11) $$u_{h}-u_{c}=-\frac{1}{k^{2}}\frac{d\varepsilon_{c}}{dx}$$

where *k* can be expressed as follows:

(12) $$k=\sqrt{\frac{\pi -2\alpha }{\pi r_{c}{}^{2}E_{c}\left[\frac{1}{G_{a}}\ln\frac{r_{a}}{r_{n}}+\frac{1}{G_{n}}\ln\frac{r_{n}}{r_{n-1}}+\ldots \frac{1}{G_{2}}\ln\frac{r_{2}}{r_{1}}+\frac{1}{G_{1}}\ln\frac{r_{1}}{r_{c}}\right]}}$$

Since the strain in the axial direction represents the first-order derivative of the axial displacement with respect to *x*, Equation (11) can be converted into:

(13) $$\frac{d^{2}\varepsilon_{c}}{dx^{2}}-k^{2}\varepsilon_{c}=-k^{2}\varepsilon_{h}(x)$$

where $\varepsilon_{h}(x)$ denotes the strain distribution in the *x*-direction on the host’s surface.

Equation (13) represents a typical second-order nonhomogeneous differential equation with constant coefficients. Its solution gives:

(14) $$\varepsilon_{c}=C_{1}\sinh(kx)+C_{2}\cosh(kx)+\varepsilon_{t}(x)$$

where *C*_1_ and *C*_2_ are the integration constants of the general solution and can be obtained in light of the boundary conditions; $\varepsilon_{t}(x)$ indicates the particular solution of Equation (13).

The fiber core strain distribution divided by the strain of the monitored host equals the strain transfer coefficient of the surface-bonded sensing cable, denoted z(x):

(15) $$z(x)=\frac{\varepsilon_{c}}{\varepsilon_{h}}$$

## 3. Analytical Solutions under Different Strain Fields

Under various loads, the strain distribution of the monitored structure can come in different forms. In this study, several typical strain field patterns were selected for analysis.

### 3.1. Linear Strain Fields

#### 3.1.1. Uniform Strain Field

**Figure 2:**
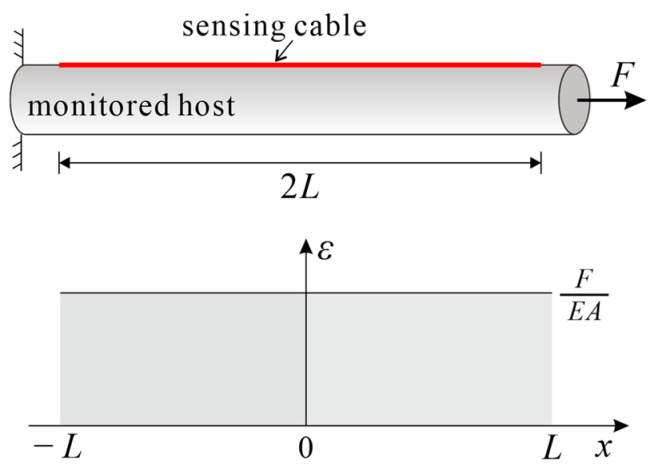
Schematic diagram of a uniform strain field.

In the monitored structure with a uniform cross-section, when one end is fixed and the other compressed or stretched, as shown in Figure 2, the strain distribution of the structure can be expressed by Equation (16).

(16) $$\varepsilon_{h}(x)=\frac{F}{EA}$$

where *E* and *A* are the Young’s modulus and cross-sectional area of the monitored host, respectively.

As the strain transfer coefficient at both ends of the cable is assumed to be 0, the boundary conditions can be expressed as:

(17) $$\varepsilon_{c}(\pm L)=0$$

In light of the boundary condition of Equation (17), the strain distribution of the fiber core is:

(18) $$\varepsilon_{c}(x)=\varepsilon_{h}(x)\left[1-\frac{\cosh(kx)}{\cosh(kL)}\right]$$

According to Equation (15), the strain transfer coefficient of the cable under uniform strain can be expressed as:

(19) $$z(x)=1-\frac{\cosh(kx)}{\cosh(kL)}$$

**Figure 3:**
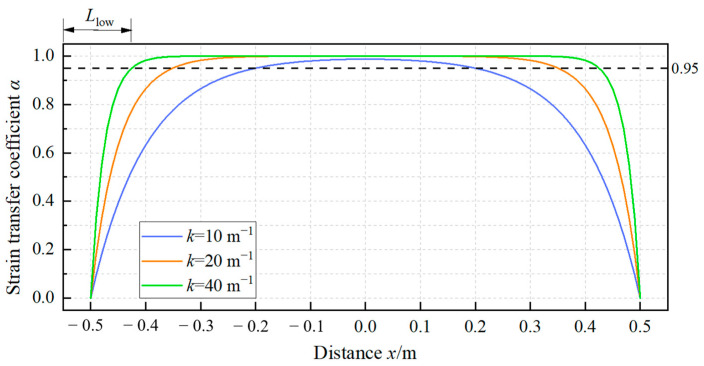
Strain transfer coefficients of the cable in the uniform strain field.

It is evident from Equation (19) that in a uniform strain field, the strain transfer coefficient of the sensing cable is independent of the magnitude of the host strain yet under the influence of the shear lag coefficient *k*. Here, the cable length was set at 1 m, the monitored host strain at 1000 με, and *k* at 10 m^−1^, 20 m^−1^, and 40 m^−1^, respectively, and the curves indicating corresponding strain transfer coefficients are presented in Figure 3. Concurring with the research findings documented in the paper in [27], the results revealed that the strain transfer coefficient curve of the entire cable exhibited a symmetrical saddle-shaped profile, with the coefficient approaching 1 in the cable’s middle section while diminishing rapidly toward 0 on both ends. In our research, the segment of the cable extending from one endpoint to where the strain transfer coefficient first reached 0.95 was denoted by the low-strain sensing segment, with its length expressed as *L*_low_. Notably, a higher shear lag coefficient, which was associated with the cable and the adhesive layer, corresponded to a reduced *L*_low_, indicative of improved strain transfer performance of the cable.

#### 3.1.2. Single-Linear Gradient Strain Field

**Figure 4:**
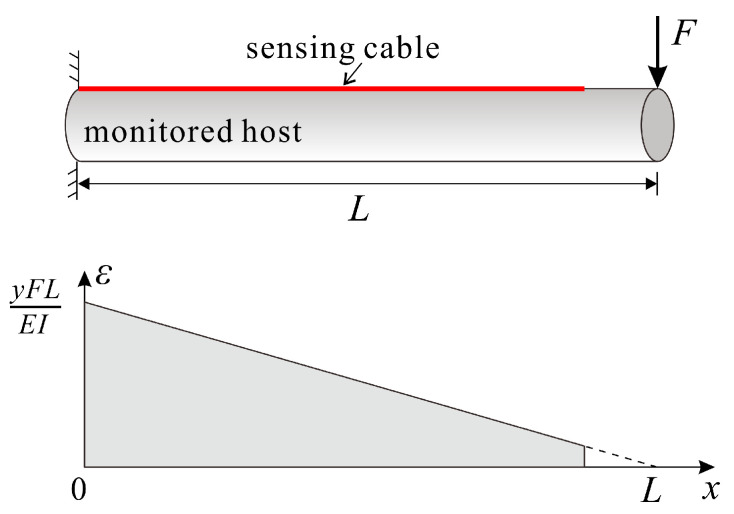
Schematic diagram of a single-linear gradient strain field.

When the monitored structure is a cantilever beam, and the non-supported end is subjected to a concentrated load, the strain distribution in the host exhibits a single-linear gradient profile, as shown in Figure 4. The expression for this strain distribution is:

(20) $$\varepsilon_{h}(x)=-\frac{yF}{EI}x+\frac{yFL}{EI}$$

where *I* is the moment of inertia of the monitored host; *y* represents the distance between the cable’s bonded area and the neutral plane.

Equation (20) conforms to the standard form for linear equations, and can be reduced to:

(21) $$\varepsilon_{h}(x)=ax+b$$

Assuming that the strain on both ends of the cable is 0, as in the case of the uniform strain field, and in accordance with the boundary condition associated with Equation (17), we obtained the expression for the strain transfer coefficient in a single-linear gradient strain field:

(22) $$z(x)=1-\frac{1}{ax+b}\left[\frac{aL\sinh(kx)}{\sinh(kL)}+\frac{b\cosh(kx)}{\cosh(kL)}\right]$$

**Figure 5:**
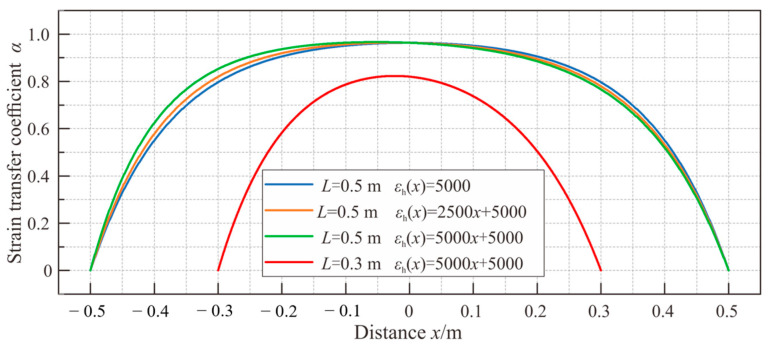
Strain transfer coefficients of the cable in single-linear gradient strain fields.

Equation (22) can be reduced to the form of Equation (19) when a is equal to 0 and b is not, in a way that depicts the strain transfer performance under uniform strain. Similarly, a simplistic calculation can be performed to compare the impacts of different strain distributions within a monitored host on strain measurements in the sensing cable. Assuming the host strain distribution $\varepsilon_{h}=5000$, $\varepsilon_{h}=2500x+5000$, and $\varepsilon_{h}=5000x+5000$, respectively, and the cable’s shear lag coefficient *k* = 6 m^−1^, the resulting curves indicating the strain transfer are displayed in Figure 5. From the calculation results, it can be observed that when the shear lag coefficient is comparatively small, the strain transfer coefficient curve of the cable in a single-linear gradient strain field differs from that in a uniform strain field. Moreover, as the slope a of the host strain distribution function increases, *L*_low_ decreases on the side under less host strain, but gradually increases on the other side. Hence, when there is a short bonding length or a small shear lag coefficient, it is imperative to analyze the strain transfer performance of the sensing cable and take into account the actual distribution of the host strain.

### 3.2. Nolinear Strain Fields

#### 3.2.1. Parabolic Strain Field

**Figure 6:**
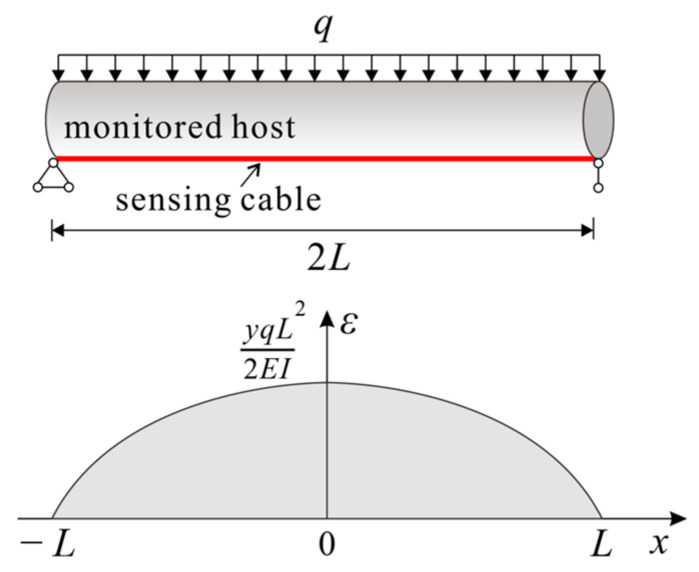
Schematic diagram of a parabolic strain field.

In the case of a simply supported beam where one side is subjected to a uniformly distributed load, the function for the host strain follows a parabolic profile, as illustrated in Figure 6. Its corresponding expression is as follows:

When a sensing cable is bonded to the loading side of the monitored host and the boundary condition of Equation (17) holds, the strain transfer coefficient of the cable is:

(23) $$z(x)=1-\frac{2}{(-x^{2}+L^{2})k^{2}}\left(1-\frac{\cosh(kx)}{\cosh(kL)}\right)$$

It is justified from Equation (23) that the strain transfer of the cable in a parabolic strain field is irrelevant to *q*, but is mainly influenced by the shear lag coefficient *k*.

#### 3.2.2. Bilinear Gradient Strain Field

**Figure 7:**
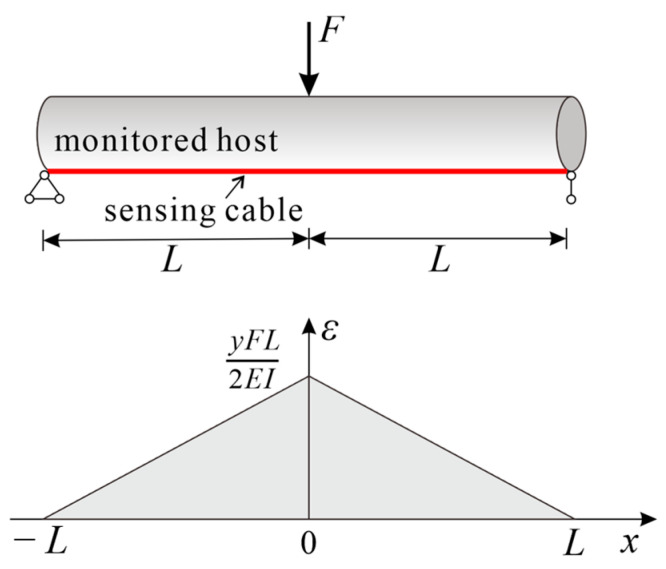
Schematic diagram of a bilinear gradient strain field.

In the multi-point bending test of uniform cross-section structures, the host strain distribution curve typically exhibits a continuous piecewise-linear shape. A three-point bending experiment, the simplest test of the kind, was exemplified in this section. Specifically, a sensing cable was bonded to the bottom surface of the host, as shown in Figure 7, with a point load applied at the midpoint on the host surface. As such, the host strain distribution can be expressed as:

(24) $$\varepsilon_{h}(x)=\frac{yF}{EI}\left(-\left|x\right|+L\right)$$

Let $a=\frac{yF}{EI}$ and $b=\frac{yFL}{EI}$, then Equation (24) can be reduced to:

(25) $$\varepsilon_{h}(x)=\left\{\begin{array}{c} \begin{array}{cc} ax+b & L_{1}\leq x<0 \end{array}\\ \begin{array}{cc} -ax+b & 0\leq x\leq L_{2} \end{array} \end{array}\right.$$

The strain and shear stress of the fiber core can be expressed, respectively, as:

(26) $$\varepsilon_{c}(x)=\left\{\begin{array}{c} \begin{array}{cc} C_{1}\cosh(kx)+C_{2}\sinh(kx)+ax+b & -L_{1}\leq x<0 \end{array}\\ \begin{array}{cc} C_{3}\cosh(kx)+C_{4}\sinh(kx)-ax+b & 0\leq x>L_{2} \end{array} \end{array}\right.$$

(27) $$\tau_{g}(x)=\left\{\begin{array}{c} \begin{array}{cc} \frac{-E_{g}r_{g}}{2}\left[kC_{1}\sinh(kx)+kC_{2}\cosh(kx)+a\right] & -L_{1}\leq x<0 \end{array}\\ \begin{array}{cc} \frac{-E_{g}r_{g}}{2}\left[kC_{3}\sinh(kx)+kC_{4}\cosh(kx)-a\right] & 0\leq x\leq L_{2} \end{array} \end{array}\right.$$

where $C_{1}$, $C_{2}$, $C_{3}$, and $C_{4}$ are coefficients to be determined by the boundary conditions of the equations. Given the spatial continuity of the optical fiber, it is assumed that the strain in the fiber core and the shear stress on the outer surface of the fiber core are continuous at the inflection point. Therefore, the boundary conditions for Equations (26) and (27) can be expressed as follows:

(28) $$\left\{\begin{array}{c} \varepsilon_{c}(\pm L)=0\\ \varepsilon_{c}(0^{-})=\varepsilon_{c}(0^{+})\\ \tau_{c}(0^{-})=\tau_{c}(0^{+}) \end{array}\right.$$

Solving the equation set gives the to-be-determined coefficients:

(29) $$\left\{\begin{array}{c} C_{1}=-\frac{a}{k}\tanh(kL)\\ C_{2}=-\frac{a}{k}\\ C_{3}=C_{1}\\ C_{4}=\frac{a}{k} \end{array}\right.$$

**Figure 8:**
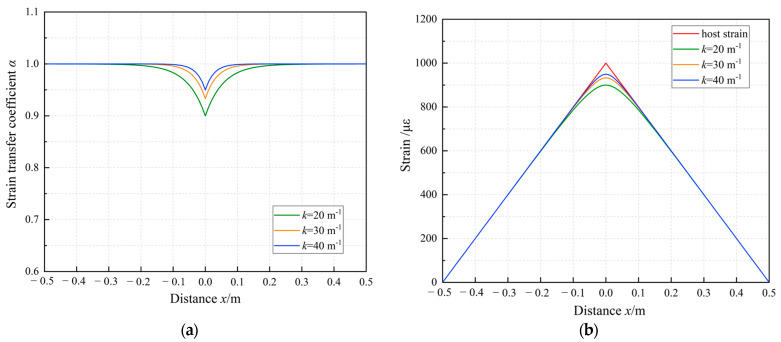
Strain transfer coefficients and fiber core strain distributions of the cable in the bilinear gradient strain field. (**a**) Strain transfer coefficient; (**b**) Fiber core strain.

By substituting the determined coefficients into Equation (26) and using Equation (15), the distribution of the strain transfer coefficient of the cable can be obtained. Assuming a bonded length of 2*L* = 1 m for the sensing cable and setting the shear lag coefficient at 20 m^−1^, 30 m^−1^, and 40 m^−1^, respectively, and with the host strain distribution $\varepsilon_{h}(x)=-2000\left|x\right|+1000$, the resulting strain transfer coefficient curve for the cable and the strain distribution in the fiber core are illustrated in Figure 8. The computational results revealed that a certain amount of strain loss occurred at the inflection point of the sensing cable in the bilinear gradient strain field. In the cable installation process, the extent of this loss was inversely correlated with the magnitude of the shear lag coefficient *k*. When *k* = 40 m^−1^, the strain transfer coefficient at the inflection point exceeded 0.95, and noticeable *L*_low_ values at both cable ends were elusive.

While the simplest form of the bilinear gradient strain field was considered in our study, practical applications involve more intricate complexities, such as asymmetric line segments or non-zero host strain at cable endpoints. In these cases, however, the conceptual framework outlined in this section can be adapted for derivation.

## 4. Experimental Validation

### 4.1. Test Setup and Procedure

**Figure 9:**
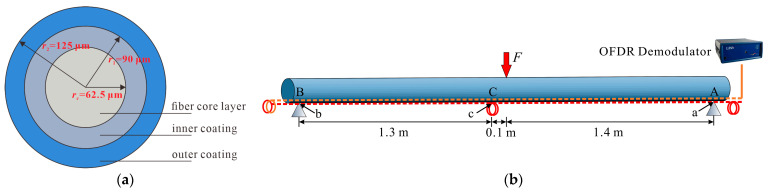
Test materials and setup. (**a**) Cross-section of the fiber; (**b**) Overall Layout.

To validate the reliability of the theoretical model and relevant conclusions, a three-point bending test was conducted on a poly vinyl chloride (PVC) pipe with a length of 3 m and an outer diameter of 7.5 cm. The strain-sensing fibers (Corning^®^ SMF-28e+^®^ LL single mode fiber) were bonded on the upper and lower surfaces of the pipe using epoxy resin. The structure of the fiber was shown in Figure 9a. The OFDR demodulator employed in the test was the 4413 optical backscatter reflectometer (OBR) developed by Luna Innovations. The measurement range of the instrument was set at 70 m, with a spatial resolution of 1 cm and a strain measurement accuracy of ±5 με [37].

The experimental setup layout is depicted in Figure 9. To be specific, two FO cables, denoted cable AB and cable ab, respectively, were bonded in parallel to the surface of the PVC tube using epoxy resin. Cable ab had a redundant section positioned 0.1 m to the left of the loading point. The cables bonded to the PVC tube faced downward, with both ends of the tube fixed using a hinged support mechanism. A weight, which involved six levels in the experiment, was suspended at the midpoint of the tube to induce deformation. The first level was subjected to a weight of 3 kg; each of the second to fourth levels underwent a progressive increment of 3 kg per level; and in the case of the fifth and sixth levels, there was a further increase of 6 kg per level. Following the load stability at each tier, strain data pertaining to the cable were gathered through the OFDR demodulator.

### 4.2. Test Results and Analysis

**Figure 10:**
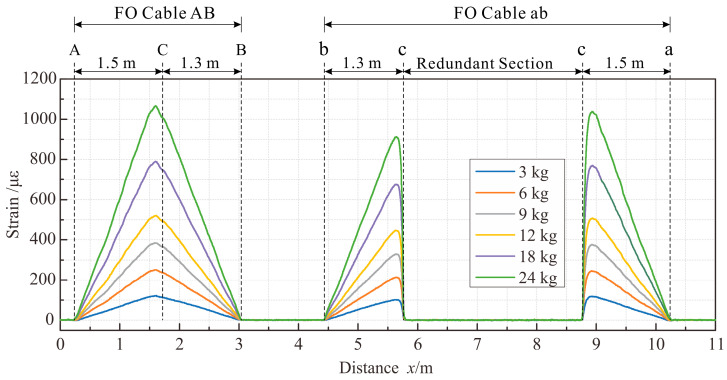
Strain distributions along the FO cable.

The strain distribution of the cable under each load is illustrated in Figure 10. The strain distribution of cable AB exhibited a symmetric triangular shape, with the maximum strain occurring at the same position as the loading point. On the other hand, cable ac can be visually divided into cable bc, cable ac, and a redundant section.

**Figure 11:**
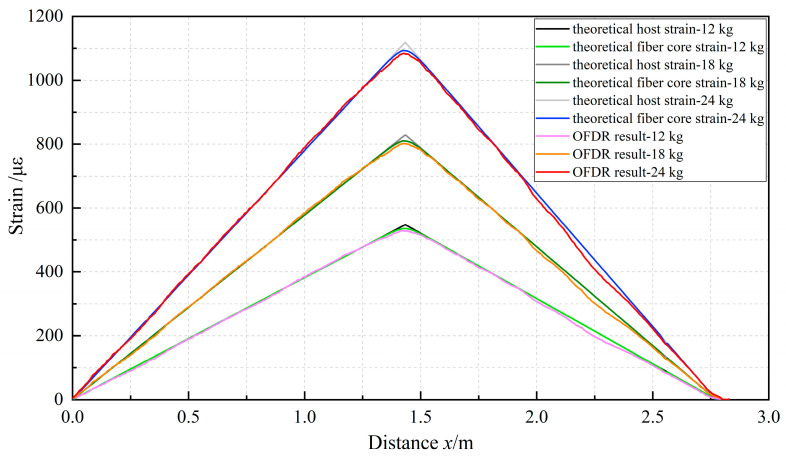
Comparison between theoretical and experimental results of cable AB.

The theoretical strain distribution of the PVC tube under three-point bending represents a typical bilinear gradient strain field. According to the solution in Section 3.2.2, the strain transfer coefficient of the sensing cable was calculated. Multiplying this coefficient by the theoretically predicted strain distribution on the surface of the PVC tube yielded the anticipated strain distribution within the fiber core. A comparison between the theoretical strain distribution of the fiber core in Cable AB cable and the OFDR measurements is depicted in Figure 11. The material composition and parameter selection for the sensing cable and the adhesive layer employed in the calculations are presented in Table 1, with the parameter values referenced from existing studies [25,27,35,38,39].

The theoretical strain of the fiber core in cable AB closely corresponded to the experimental results, validating the applicability of the proposed theoretical model to the analysis of strain transfer in the cable under a bilinear gradient strain field. Within a range of 10 cm on either side of the inflection point, the cable exhibited a discernible strain loss. In the proximity of the inflection point, the measured strain values were slightly below theoretical calculations and featured noticeable fluctuations, which were possibly attributed to uneven adhesive bonding and equipment system errors. Additionally, the measurements from OFDR aligned with the theoretical values of strain on the PVC tube surface. This indicated that within these ranges, the strain transfer performance of the sensing cable is highly reliable, showing that the fiber core strain can accurately reflect the monitored host strain.

**Figure 12:**
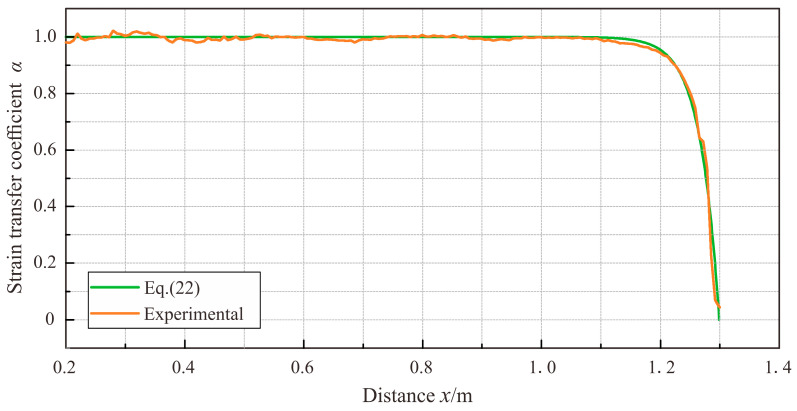
Comparison between theoretical and experimental results of cable bc.

The host strain distribution of the 1.3 m-long cable bc followed the patterns of a single-linear gradient strain field. Consistent with the theoretical analysis results presented in Section 3.1.2, at the non-supported end c of cable bc, the strain of the cable was seen to plummet down to 0, indicative of the emergence of a low-strain sensing segment. The aforementioned results suggested that except for a range of 10 cm on either side of the cable, the strain distribution of cable AB can be regarded as the actual strain on the surface of the PVC tube. Therefore, the strain in cable bc divided by that at the corresponding position in cable AB is the measured strain transfer coefficient of cable bc. With Equation (22) from Section 3.1.2, the theoretical strain transfer coefficient was calculated. The comparison between the theoretical calculations and measured results of the strain transfer coefficient of the sensing cable under the sixth load is presented in Figure 12. The theoretical calculations of the strain transfer coefficient of cable bc were consistent with experimental results, indicating that the derived strain transfer coefficient of linear strain fields, as presented in this study, is applicable to analyzing the strain transfer performance of sensing cables. In practical applications, the strain transfer coefficient of sensing cables can be theoretically calculated with merely the parameters of the cables and their adhesive layers available.

## 5. Parametric Analysis

With an aim to offer insights into the design and installation of DFO sensors, a parametric analysis based on Equation (22) was undertaken in this section, where the effects of mechanical and geometric properties of the protective and adhesive layers in the FO cable on the strain transfer of the fiber optic sensor were examined. The host strain distribution was set at $\varepsilon_{h}(x)=1000x+1000$ and the length of the bonded cable at 1 m, using the parameter values specified in Table 1. When the impact of a particular parameter was analyzed, all others remained unchanged.

**Figure 13:**
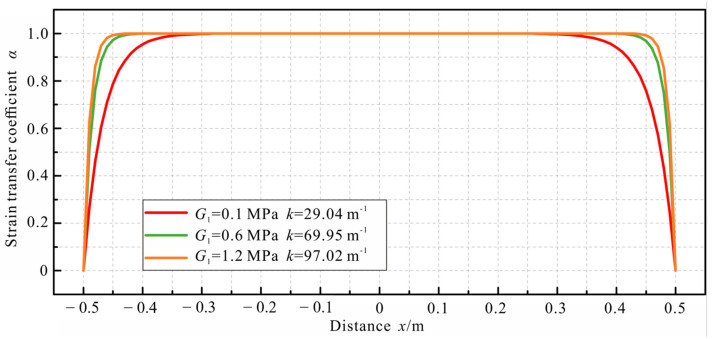
Impact of the shear modulus of the inner coating on the strain transfer coefficient.

Firstly, the impact of the shear modulus *G*_1_ of the inner coating layer on the strain transfer coefficient was analyzed. The shear modulus was set at 0.1 MPa, 0.6 MPa, and 1.2 MPa, respectively. The distribution of strain transfer coefficients of the bonded cable, calculated using Equation (22), is shown in Figure 13. From the calculation results, it was evident that the shear modulus of the inner coating *G*_1_ had a significant influence on the strain transfer performance of the cable. As *G*_1_ increased, the low-strain sensing segment diminished, resulting in a considerable enhancement in the strain transfer performance of the cable. Thus, when fabricating sensing cables, it is advisable to opt for coating materials with a higher shear modulus to mitigate any adverse effects on the strain transfer performance.

Similarly, an analysis was conducted to assess the impact of the shear modulus of the outer coating on the strain transfer performance of the cable. The shear modulus of the outer coating *G*_2_ varied at 50 MPa, 600 MPa, and 1200 MPa, yielding corresponding shear lag coefficients *k* of 31.79 m^−1^, 31.81 m^−1^, and 31.81 m^−1^, respectively. Analogous to the influence of the shear modulus of the inner coating, a higher shear modulus of the outer coating contributed to the improved strain transfer performance of the cable. However, the impact of *G*_2_ on the strain transfer performance was relatively minor, as exemplified by the nearly identical outcomes produced with the shear modulus at 600 MPa and 1200 MPa. This observation suggests that the outer coating and other external protective layers can effectively safeguard the cable without significantly compromising its strain transfer performance.

Lastly, an investigation was carried out into the influence of the shear modulus *G*_a_ of the adhesive layer and the adhesive thickness *t*. The shear modulus of the adhesive layer was set at 2.9 MPa, 29 MPa, and 290 MPa, resulting in corresponding shear lag coefficients of 30.88 m^−1^, 31.79 m^−1^, and 31.88 m^−1^, respectively. This demonstrated that a higher shear modulus of the adhesive layer led to an increased shear lag coefficient, thereby improving the strain transfer performance of the cable, albeit in a limited manner. Alternatively, when the adhesive thickness was set at 20 μm, 200 μm, and 2000 μm, the shear lag coefficients were calculated to be 32.00 m^−1^, 31.79 m^−1^, and 31.47 m^−1^, respectively. It was evident that as the thickness of the adhesive layer between the cable and the monitored host increased, the strain transfer performance of the cable decreased. However, similar to the shear modulus, the impact of adhesive thickness on the strain transfer performance was modest. Given that, to enhance the strain transfer performance of the sensing cable during installation, it is recommended to use an adhesive with a higher shear modulus that ensures close adherence of the cable to the host surface. Additionally, considering the protective function of the adhesive, an increase in adhesive thickness can provide additional safeguarding for the cable. Likewise, in designing and manufacturing the cable, enhancing the shear modulus of the protective layers and moderately increasing their thickness can make the cable more resistant to challenging environmental conditions while maintaining outstanding strain transfer performance. In real-world applications, a rigid jacket may diminish the peak strain detection capability of the sensing cable. Hence, it is crucial to maintain the shear modulus of the jacket material within a reasonable range.

## 6. Conclusions

In this paper, we established a strain transfer model for the surface-bonded DFO sensors with multilayered structures as a way of inferring the strain transfer coefficient of the sensor when the strain of the monitored host was distributed in uniform, parabolic, single-linear gradient, and bilinear gradient manners. An investigation was systematically conducted into how the monitored structure’s strain field impacted the strain measurements of DFO sensors, followed by experimental validation. The conclusions are as follows:

- The strain distribution of the monitored host had an effect on the strain transfer of the DFO sensor. In the linear strain field, there was a noticeable low-strain segment at both ends of the bonded cable area, and the strain transfer performed well across the cable, except for the low-strain sensing segment. As the shear lag coefficient *k* of the cable decreased, the length of the low-strain sensing section *L*_low_ increased. In applications, the ideal length of the deployed cable should exceed 2*L*_low_. In the case of a shorter cable length or a small shear lag coefficient, it is necessary to review the strain transfer mechanism of the sensing cable in a way that examines and rectifies errors from the system for measuring the strain in DFO sensors.
- In the bilinear gradient strain field, the strain loss of the sensing cable occurred at the inflection point of the monitored host’s strain distribution, albeit with limited influence. Notably, when the shear lag coefficient *k* reached 40 m^−1^, the strain transfer coefficient of the bonded cable segment consistently exceeded 0.95. However, in scenarios where the equipment’s spatial resolution is high and the dimensions of the monitored host are small, careful consideration should be given to the impact of strain loss at the inflection point on the accuracy of strain measurements.
- The findings from the parametric analysis revealed that the shear modulus of protective layers, such as the coatings and jackets, was positively correlated with the shear lag coefficient k and strain transfer performance of the cable. Additionally, such performance was most influenced by the shear modulus of the low-strength inner coating and moderately impacted by that of the outer protective layer. Furthermore, the radius of the jacket and adhesive layer inversely affected the shear lag coefficient *k* of the cable, resulting in a reduction in its strain transfer performance. However, the magnitude of this impact was relatively minor. When engaging in surface bonding, it is advisable to position the cable in close proximity to the surface of the to-be-monitored host.
- In practical applications, it is advisable to opt for coating materials with a higher shear modulus to mitigate adverse impacts on strain transfer in the cable. An increase in the shear modulus of the jacket and other protective layers can, to a limited extent, improve the strain transfer performance of the cable. However, an excessive shear modulus of the jacket compromises the cable’s capability to measure peak strain, which thus necessitates the control of the jacket shear modulus within a reasonable range. Although thicker protective layers reduce the strain transfer performance of the cable, the extent of this influence is limited. Therefore, appropriately increasing the thickness of the protective layers can enhance their resilience against harsh engineering environments.
- The results in this paper are based on several simple strain forms, and preliminarily reveal the influence of strain distribution on the strain transfer of DFO sensors, which provides a theoretical foundation. However, in practical engineering, the strain field of the structure is difficult to describe using simple expressions, and the strain transfer of DFO sensors in this issue needs to be further explored.

## Figures and Tables

**Table 1:** The materials and parameters of the FO cable and the adhesive layer.

Component	Material	Parameter	Value	Unit
Fiber core	Silica	Radius *r_c_*	62.5	μm
		Young’s modulus *E*_c_	72	GPa
Inner coating	Acrylate (soft)	Radius *r*_1_	95	μm
		Shear modulus *G*_1_	0.05	MPa
Outer coating	Acrylate (hard)	Radius *r*_2_	125	μm
		Shear modulus *G*_2_	0.5	MPa
Adhesive	Epoxy resin	Thickness *t*	200	μm
		Shear modulus *G*_a_	29	MPa

## Data Availability

The datasets for this study are available from the corresponding author upon reasonable request.
